# The Effect of Plasma Triglyceride-Lowering Therapy on the Evolution of Organ Function in Early Hypertriglyceridemia-Induced Acute Pancreatitis Patients With Worrisome Features (PERFORM Study): Rationale and Design of a Multicenter, Prospective, Observational, Cohort Study

**DOI:** 10.3389/fmed.2021.756337

**Published:** 2021-12-13

**Authors:** Longxiang Cao, Jing Zhou, Mingzhi Chen, Tao Chen, Man Liu, Wenjian Mao, Jiyan Lin, Donghuang Hong, Weijie Yao, Yi Sun, Kaixiu Qin, Feng Guo, Yun Zhou, Qinghai Jiao, Yingjie Chen, Gang Li, Bo Ye, Lu Ke, Zhihui Tong, Yuxiu Liu, Weiqin Li, Chinese Acute Pancreatitis Clinical Trials Group (CAPCTG)

**Affiliations:** ^1^Department of Critical Care Medicine, Jinling Hospital, Medical School of Nanjing University, Nanjing, China; ^2^Department of Critical Care Medicine, Jinling Hospital, Nanjing Medical University, Nanjing, China; ^3^Department of Critical Care Medicine, Jinjiang Hospital of Traditional Chinese Medicine, Jinjiang, China; ^4^Department of Public Health, Policy and Systems, Institute of Population Health, The University of Liverpool, Liverpool, United Kingdom; ^5^National Institute of Healthcare Data Science, Nanjing University, Nanjing, China; ^6^Emergency Department, The First Affiliated Hospital of Xiamen University, Xiamen, China; ^7^Department of Critical Care Medicine, Fujian Provincial Hospital, Fuzhou, China; ^8^Department of Hepatobiliary Surgery, General Hospital of Ningxia Medical University, Yinchuan, China; ^9^The Fourth Department of The Digestive Disease Center, Suining Central Hospital, Suining, China; ^10^Department of Emergency Medicine, The Second Affiliated Hospital of Chongqing Medical University, Chongqing, China; ^11^Department of Intensive Care Unit, Sir Run Run Shaw Hospital of Zhejiang University School of Medicine, Hangzhou, China; ^12^Department of Critical Care Medicine, Pingxiang People's Hospital, Pingxiang, China; ^13^Department of Critical Care Medicine, The First Hospital of HanDan, Handan, China; ^14^Department of Biostatistics, School of Public Health, Southern Medical University, Guangzhou, China

**Keywords:** acute pancreatitis (AP), hypertriglyceridemia (HTG), TG-lowering therapy, organ failure free day, cohort study

## Abstract

**Background:** Acute pancreatitis (AP) is a potentially life-threatening inflammatory disease with multiple etiologies. The prevalence of hypertriglyceridemia-induced acute pancreatitis (HTG-AP) has been increasing in recent years. It is reported that early triglyceride (TG) levels were associated with the severity of the disease, and TG- lowering therapies, including medical treatment and blood purification, may impact the clinical outcomes. However, there is no consensus regarding the optimal TG-lowering therapy, and clinical practice varies greatly among different centers. Our objective is to evaluate the TG-lowering effects of different therapies and their impact on clinical outcomes in HTG-AP patients with worrisome features.

**Methods:** This is a multicenter, observational, prospective cohort study. A total of approximately 300 patients with HTG-AP with worrisome features are planned to be enrolled. The primary objective of the study is to evaluate the relationship between TG decline and the evolution of organ failure, and patients will be dichotomized depending on the rate of TG decline. The primary outcome is organ failure (OF) free days to 14 days after enrollment. Secondary outcomes include new-onset organ failure, new-onset multiple-organ failure (MOF), new-onset persistent organ failure (POF), new receipt of organ support, requirement of ICU admission, ICU free days to day 14, hospital free days to day 14, 60-day mortality, AP severity grade (Based on the Revised Atlanta Classification), and incidence of systemic and local complications. Generalized linear model (GLM), Fine and Gray competing risk regression, and propensity score matching will be used for statistical analysis.

**Discussion:** Results of this study will reveal the current practice of TG-lowering therapy in HTG-AP and provide necessary data for future trials.

## Background

Acute pancreatitis (AP) is a potentially life-threatening inflammatory disease caused by multiple etiologies, such as alcohol, gallstones, and hypertriglyceridemia (HTG). HTG is the third most common cause of AP, accounting for 4–10% of cases globally, and the increasing prevalence of HTG-AP had been reported in recent studies (1–4). In China, HTG had been the second leading cause of AP, and previous studies showed that HTG-AP patients had a higher risk of severe acute pancreatitis and multiple organ dysfunction syndrome (MODS) than other types of AP (2, 5–7).

Although the pathophysiology underlying HTG-AP remains controversial, it is widely accepted that free fatty acid (FFA) is one of the driving factors (8). FFA, produced by the hydrolysis of triglyceride (TG), can initiate or worsen the disease by triggering inflammatory reactions, damaging the pancreatic cell, and promoting microvascular thrombosis within the pancreatic tissue (9). Nawaz et al. (6) found that elevated serum TG levels in AP patients were independently and proportionally correlated with persistent organ failure (POF) regardless of etiology. In an observational study conducted by Lu et al. (10) timely reduction of serum TG during the early phase of HTG-AP was found to be associated with decreased incidence of POF.

Over the past years, several attempts had been made to lower serum TG more efficiently during the acute phase of the disease, including medical treatment with insulin and/or heparin, blood purification, and gene therapy in cases (11). Medical treatment is convenient and safe and is considered the first-line choice for TG-lowering therapy (4). Heparin stimulates the release of endothelial lipoprotein lipase into circulation, while insulin activates lipoprotein lipase, thereby increasing the clearance of chylomicrons from plasma (12). However, the impact of medical therapy on clinical outcomes is uncertain, and an observational study is ongoing to figure it out (13). Blood purification, especially plasmapheresis, is also widely used in TG-lowering therapy. Plasmapheresis rapidly removes triglycerides from plasma and is considered one of the most efficient TG-lowering therapies (14). Technically, it is a therapeutic procedure in which the blood of the patient is passed through a medical device that separates plasma from other components of blood. The plasma is removed and replaced by a replacement solution (e.g., albumin and/or plasma) or a combination of crystalloid/colloid solution (15). Double filtration plasmapheresis (DFPP) is a semi-selective apheresis method based on a double filter system, which can remove macromolecules selectively (16). Both techniques are widely adopted, while plasmapheresis is thought to be more effective in removing FFA (17). Other blood purification modalities were also reported effective in lowering plasma TG, including hemoperfusion and hemofiltration (18, 19). A randomized control trial (RCT) reported that high-volume hemofiltration (HVHF) decreased TG levels more efficiently than medical therapy (18).

For the target of TG-lowering therapy, it is regarded that reducing the TG level to 5.65 mmol/L might be clinically sufficient (20). Lu et al. (10) found that patients with earlier TG levels of < 5.65 mmol/L were less likely to develop POF. However, the optimal TG lowering target and choice of therapies in early HTG-AP are unclear due to the lack of high-quality studies. Given the paucity of evidence in the literature and the variation in the management of HTG-AP, we conducted this multicenter, observational study and built “The effect of plasma triglyceride-lowering therapy on the evolution of organ function in early hypertriglyceridemia-induced acute pancreatitis patients with worrisome features”(PERFORM) registry to evaluate the TG-lowering effects of different therapies and their impact on clinical outcomes in HTG-AP patients with worrisome features.

## Methods

### Study Objectives

The primary objective of the study is to evaluate the relationship between TG decline and the evolution of organ failure in a cohort of early HTG-AP patients with worrisome features. The secondary objectives are to characterize the current clinical practice regarding TG-lowering therapy, describe the association between different choices of therapy and clinical outcomes. The third objective is to provide data for future trials. Accordingly, every secondary endpoint is also supposed to be hypothesis generating.

### Study Design

The PERFORM study is a multicenter, observational, prospective cohort study. The overall cohort is HTG-AP patients presenting with worrisome features. It was registered on October 30th, 2020, in the Chinese Clinical Trial Registry (ChiCTR2000039541, https://www.chictr.org.cn/index.aspx). The PERFORM study was designed and coordinated by the Chinese Acute Pancreatitis Clinical Trials Group (CAPCTG).

### Study Population

Recruitment started on November 1st, 2020, and is scheduled to end on October 31st, 2022. This study is planned to recruit patients admitted to 30–40 hospitals across China. All adult patients presenting with early HTG-AP with worrisome features would be consecutively enrolled. The definition for HTG-AP with worrisome features is indicated as below.

**Figure 1:**
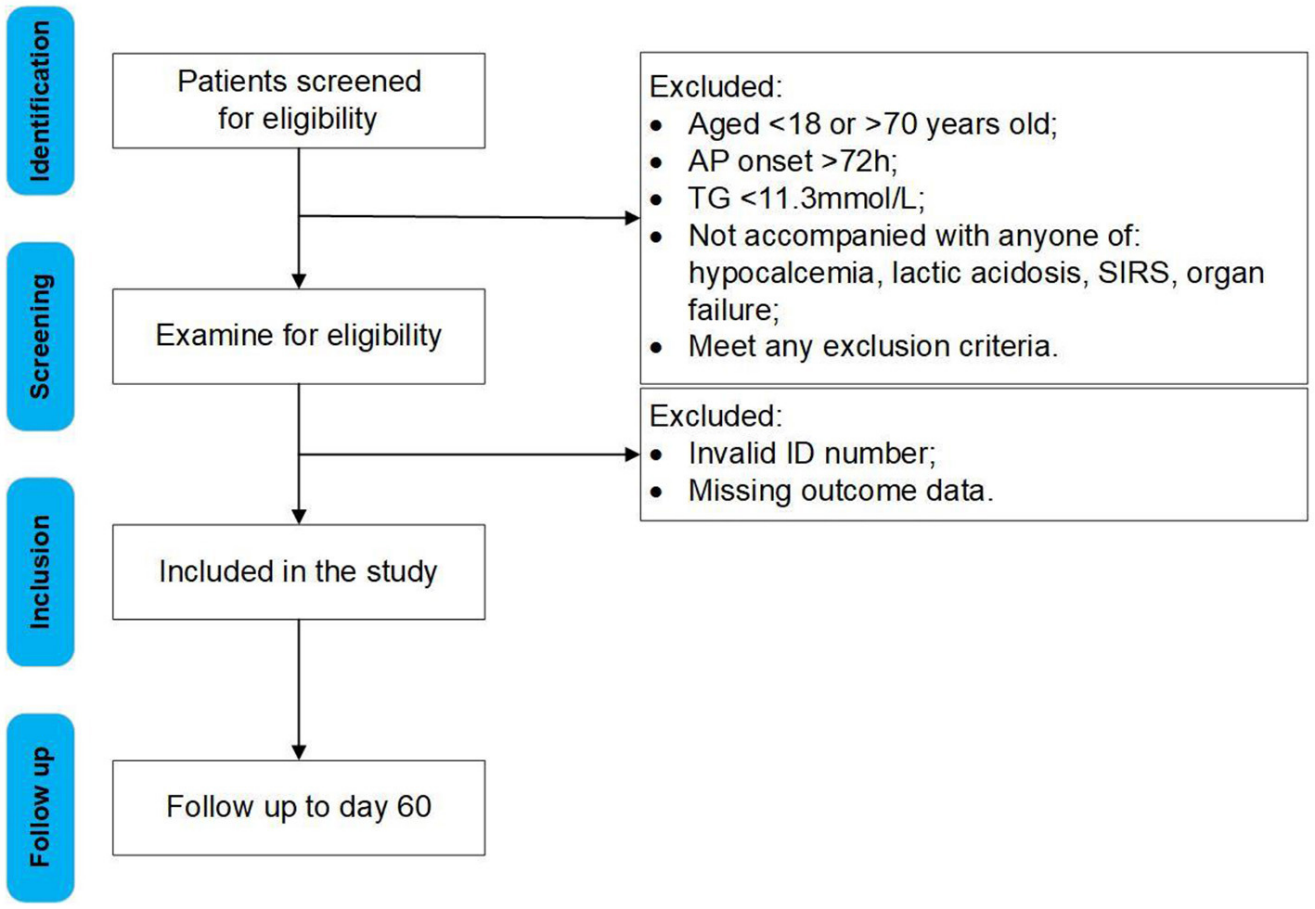
Flow chart of participants.

All adult patients with AP admitted to the participating centers will be assessed for eligibility after admission (Figure 1). The inclusion and exclusion criteria are as follows:

### Inclusion Criteria

1. Age Between 18 and 70 Years old;
2. Within 72 H From the Onset of Abdominal Pain;
3. Symptoms and Signs of AP Based on Abdominal Pain Suggestive of AP, Serum Amylase at Least three Times the Upper Limit of Normal, and/or Characteristic Findings of AP on Computed Tomography or Less Commonly Magnetic Resonance Imaging (MRI) or Transabdominal Ultrasonography According to the Revised Atlanta Criteria (21).
4. When Enrolled, TG > 1,000 mg/dL (11.3 mmol/L), Accompanied by the Clinical Features of any one or More of the Following (22):
1) Signs of hypocalcemia (calcium levels < 2 mmol/L);
2) Lactic acidosis (Lactate levels more than 2 mmol/L and PH < 7.35);
3) The systemic inflammatory response syndrome (SIRS) is clinically recognized by the presence of two or more of the following:
a) Temperature >38.5°C or <35.0°C;
b) Heart rate of >90 beats /min;
c) Respiratory rate of >20 breaths/min or PaCO2 of <32 mmHg;
d) WBC count of >12, 000 cells/mL, <4,000 cells/mL, or >10 percent immature (band) forms;
4) Organ failure defined by the sequential organ failure assessment (SOFA) score for respiration, renal and cardiovascular systems.

### Exclusion Criteria

1. Failure to Obtain Informed Consent;
2. Pregnant or Lactating Women; Or Have a Pregnancy Plan Within a Month After the Study (Including Male Subjects);
3. Researchers' Family Members who Are Directly Involved in the Study;
4. Patients Are Expected to die Within 48 H After Enrollment, Defined as Patients With Norepinephrine Usage at a Dose of 25 mg/min or More Under Full-Fluid Resuscitation, With a Systolic Blood Pressure <90 mm Hg and Serum pH Values <7.0. The Judgment Will be Made by the Treating Physician.

Informed consent should be sought for each participant or a patient's relative. They are free to withdraw from the study for any reason without the need for further explanation. This prospective observational study examines clinical outcomes in early HTG-AP patients with worrisome features treated with TG lowering therapy (from fasting to sophisticated blood purification). Therefore, no mandatory intervention or randomization is proposed. The main treatment modalities for TG-lowering therapy include fasting, medical treatment (either heparin, insulin, or both), and blood purification, including hemoperfusion, hemofiltration, and therapeutic plasmapheresis. The treatment is at the discretion of treating physicians.

We recommend all patients receive standard treatment that follows the “Acute Pancreatitis Treatment Guidelines” issued by the American College of Gastroenterology (ACG) in 2013 and the standard treatment plan for acute pancreatitis provided in the “Evidence-Based Guidelines for the Treatment of Acute Pancreatitis” issued by the International Association of Pancreatology (IAP) and the American Pancreatic Association (APA) (23, 24).

### Primary Outcome Measure

The primary outcome is organ failure (OF) free days to 14 days after enrollment. Only the final period of OF-free days is included, and patients who have OF at day 14 or died before day 14 are assigned to zero OF-free days.

Secondary outcome measures

Part I: Secondary outcomes during the index admission

1. New-onset organ failure;
2. New-onset multiple-organ failure (MOF);
3. New-onset persistent organ failure (POF);
4. New receipt of organ support;
5. Requirement of ICU admission;
6. ICU free days to day 14;
7. Hospital free days to day 14;

Part II: Secondary outcomes within 60 days after enrollment

1. Mortality censored at 60 days after enrollment;
2. AP severity grade (Based on the Revised Atlanta Classification);
3. Incidence of infected pancreatic necrosis (IPN);
4. Incidence of septic shock;
5. Incidence of abdominal bleeding;
6. Incidence of gastrointestinal fistula.

### Definition of Outcomes

An individual SOFA score of 2 or more for the respiration, cardiovascular, or renal system is defined as the presence of organ failure. New-onset organ failure is defined as organ failure that is not present at any time in the 24 h after enrollment. Multiple organ failure is defined as two or more organ failures present at the same time. Persistent organ failure is defined as organ failure that persists for more than 48 h. ICU free days to day 14 after enrollment is defined as the number of days alive and not admitted to an ICU after the patient's latest discharge from the ICU before day 14. If the patient is admitted to an ICU on day 14 or dies prior to day 14, ICU-free days will be 0. Hospital-free days to day 14 after enrollment is defined as the number of days alive and not admitted to the hospital after the patient's final discharge from the hospital before day 14.

### Data Collection and Management

A web-based electronic database (access through the website of the CAPCTG, https://capctg.medbit.cn/) is used for data collection and storage. All data are de-identified and input by the primary investigator or nominated investigators (less than two for each participating center) approved by the primary investigator, and a double check will be done by the research coordinator. Training for data entry is performed by the provider of the electrical database (Unimed Scientific, Inc, Wuxi, China) and the coordinating and data management center of the CAPCTG. Data including demographic characteristics, baseline characteristics, daily laboratory test, daily TG-lowering treatment, daily SOFA score, and follow-up characteristics. Demographic characteristics include age and sex. Baseline characteristics include body mass index (BMI), SOFA score on admission, Acute Physiology and Chronic Health Evaluation II (APACHE II) score on admission, the systemic inflammatory response syndrome (SIRS) on admission. Daily laboratory tests include serum total cholesterol (TC), triglyceride (TG), high-density lipoprotein cholesterol (HDL-c), low-density lipoprotein cholesterol (LDL-c), apolipoprotein A1 (Apo A1), apolipoprotein B (Apo B), apolipoprotein E (Apo E), lipoprotein a [LP(a)], free fatty acids (FFA), C-reactive protein (CRP), and procalcitonin (PCT). Daily TG-lowering treatment includes blood purification treatment (e.g., plasma exchange, hemoperfusion, and hemofiltration) and medical treatment (e.g., insulin and heparin). Follow-up characteristics include ICU days, hospital days, in-hospital cost, revision of the Atlanta classification on admission, CT severity index (CTSI) score (Based on the last image before discharge or death), mortality, and incidence of major complications on day 60. According to the schedule shown in Table 1, the investigators are required to collect data during the index admission and on day 60 after enrollment. And a follow-up on day 60 will be implemented through telephone.

**Table 1:** Schedule of enrollment, assessment and follow up.

	**Study period**			
	**Enrollment**	**Observational period**		**Discharge**
**Time point**	<72 h	Day0^a^	Day1-Day14^b^	Day28 and Day60
**Enrollment:**				
Eligibility screen	X			
Informed consent	X			
Laboratory test	X			
Imaging(CT scan etc.)	X			
**Assessment:**				
Organ failure		X	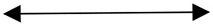	
Laboratory test		X	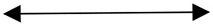	
Major treatment		X	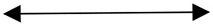	
Adverse effects		X	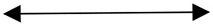	
**Follow up:**				
Vital status				X
Major complication				X
ICU days and hospital days				X
Cost				X

a *Day0 is defined as the day from enrollment to 8 am the next day*.

b *DayX is defined as the day from 8 am day X after enrollment to 8 am the next day*.

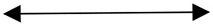
: *Assessments during this period need to be repeated on a daily basis*.

### Sample Size Justification

Based on the feasibility and patient flow of the participating sites, a sample size of 300 patients was expected, with an average of 15 patients per month within 2 years. Considering an estimated 20% rate of incomplete data or losses of follow-up, our expected sample size (240 patients) would provide 87% to detect a 2-days (SD: 5) or 82.5% for 1.5-days (SD: 4) improvement of organ failure free days between patients achieve target TG and those not.

### Statistical Analysis

Continuous normally distributed data were reported as means with SDs. Skewed continuous data were reported as medians and interquartile ranges (IQRs). Categorical data will be summarized by counts and percentages. The intergroup difference will be compared by Student's *t*-test or Wilcoxon rank-sum test for continuous variable depending on their normality and chi-square test for categorical data.

To evaluate the association between TG decline and OF free days, the study patients will be dichotomized depending on whether the TG level reaches 5.65 mmol/L on Day 3 (the day of enrollment is labeled Day 1, the next day labeled Day 2, and the following day Day 3). For the primary outcome comparison, Wilcoxon rank-sum test will be employed. However, since OF could be evaluated with a time-to-event analysis censored at 14 days to account for the mortality as a competing event, Fine and Gray competing risk regression is used to assess the group difference as a supportive analysis.

For the association between different TG lowering therapy and OF free days, we considered the possibility that baseline characteristics, which were expected to be prognostic for OF, differ according to the choice of TG lowering therapies (i.e., blood purification treatment and medical treatment). A propensity score matching will be further used to compensate for the intergroup unbalance.

For secondary outcomes, a multivariate analysis generalized linear model (GLM) model will be performed to identify its association with TG decline and TG-lowering therapy with proper link and distribution function. The variable included in the model will be age, sex, TG level at enrollment, and other baseline variables that have significant differences between groups.

## Discussion

Several studies have shown that TG level was associated with the development of organ failure in HTG-AP patients (6, 7, 10). However, few studies evaluated whether timely reduction of TG levels can impact the evolution of organ failure during the early phase of HTG-AP. A retrospective study found that patients reaching the target TG level of < 5.65 mmol/L faster were less likely to develop POF (*P* = 0.002) (10). However, considering the embedded bias of retrospective studies, prospective studies are needed to provide reliable data for future trials.

Despite the paucity of evidence, prompt reduction of triglycerides is commonly considered helpful (8, 25, 26). Given the role FFA may play in the pathophysiology of HTG-AP, insulin therapy seems promising, as it lowers TG level by reversing the stress-associated release of fatty acids from adipocytes, which can promote intracellular TG generation within adipocytes and fatty acid metabolism (27). For heparin, it stimulates the release of LPL from endothelial cells. However, its use remains controversial because the increase of serum LPL caused by heparin can decrease rapidly due to hepatic degradation, resulting in depletion in the LPL storage (13). Insulin/heparin treatment has been frequently used in the management of HTG-AP. However, its impact on TG reduction and clinical outcomes is unclear. A retrospective study conducted by Dhindsa et al. (28) showed a similar triglyceride-lowering effect between additional insulin infusion and conventional therapy. On the contrary, a meta-analysis reviewed three RCTs found intensive insulin therapy was associated with a shorter length of hospitalization and lower APACHE II score in SAP patients (29).

For blood purification, several studies assessed the effect of plasmapheresis on TG reduction and clinical outcomes. A systematic review involving eight studies found that it is effective in reducing TG level with a 69.6% decrease after treatment (14). Two studies reported a reduction of APACHE-II scores before and after plasmapheresis, while another retrospective study found plasmapheresis did not decrease morbidity or mortality (30–32). Based on the current evidence, HTG-AP is a category III, grade 2C indication for therapeutic plasma exchange in the American Society for Apheresis (ASFA) guidelines (15). Hemoperfusion (HP) is another blood purification modality that can absorb large pathogenic molecules from circulation by adsorbent materials installed in the HP cartridge. Hemofiltration is another choice, which was reported to be beneficial for AP patients (33, 34). However, there are no studies that have demonstrated the benefits of HP among HTG-AP patients by now. For other modalities, an RCT by He et al. (18) found that high-volume hemofiltration (HVHF) can lower TG levels more efficiently than insulin/heparin therapy but cannot improve clinical outcomes. A small pilot study enrolled 20 HTG-AP patients undergoing conventional treatment alone (the control group) or combined HVHF and HP treatment. The results showed a more significant reduction of TG level and improved clinical outcomes in the latter (35).

Taken together, there is no high-quality evidence demonstrating the clinical benefits of any specific TG-lowering therapy, and the primary choice of treatment varies significantly among different centers. In the 2021 Chinese guideline, TG lowering therapy, including fasting, medical treatment, and blood purification, are recommended when TG level is higher than 11.3 mmol/L. But the quality of evidence is moderate. Moreover, there are no clear recommendations regarding speficific TG-lowering therapy both in Chinese and international guidelines (23, 24, 36–38). The present study is designed to describe the current practice of TG-lowering therapy in HTG-AP and provide necessary data for future trials.

## Ethics Statement

The PERFORM study was approved by the Ethics Committee of Jinling Hospital Nanjing University (No. 2020NZKY-016-01) prior to recruitment. All the participating sites are required to obtain local ethics approval before the commencement of recruitment. The participants will provide their written informed consent to participate in this study.

## Author Contributions

WL and ZT contributed to conception and design of the study. YL and LK reviewed and revised the manuscript. LC and JZ wrote the first draft of the manuscript. TC and ML wrote the statistical analysis plan. WM participated in preparing tables and figures. MC, JL, DH, WY, YS, KQ, FG, YZ, QJ, YC, GL, and BY revised the manuscript, provided comments, and technical advice. All authors contributed to manuscript revision, read, and approved the submitted version.

## Funding

This study was funded partly by Key Research and Development Program Foundation of Jiangsu Province of China (No. BE 2016749). This study was funded partly by the National Science Foundation of China (No. 81900592).

## Conflict of Interest

The authors declare that the research was conducted in the absence of any commercial or financial relationships that could be construed as a potential conflict of interest.

## Publisher's Note

All claims expressed in this article are solely those of the authors and do not necessarily represent those of their affiliated organizations, or those of the publisher, the editors and the reviewers. Any product that may be evaluated in this article, or claim that may be made by its manufacturer, is not guaranteed or endorsed by the publisher.
