# Supplementary material for: The Effect of Plasma Triglyceride-Lowering Therapy on the Evolution of Organ Function in Early Hypertriglyceridemia-Induced Acute Pancreatitis Patients With Worrisome Features (PERFORM Study): Rationale and Design of a Multicenter, Prospective, Observational, Cohort Study
Source: Front Med (Lausanne). 2021 Dec 13;8:756337. doi: 10.3389/fmed.2021.756337 (PMC8710509; doi:10.3389/fmed.2021.756337)
Supplement: Supplementary file 1 [file Table_1.DOCX]

Supplementary Material

# Supplementary Table

Table1: Overview of adverse events related to plasmapheresis

| Overview of adverse events | |
| --- | --- |
| Replacement fluid-related complications | Hypokalemia |
|  | Hypocalcemia |
|  | Immunglobulin depletion |
|  | Coagulation factor depletion |
| Citrate-related complications | Hypocalcemia |
|  | Metabolic alkalosis |
|  | Paresthesia |
|  | Nausea |
|  | Vomiting |
|  | Chest pain |
|  | Hypotension |
|  | Tetany |
|  | Arrhythmias |
| Vascular catheter-related complications | Infection |
|  | Pain |
|  | Nerve damage |
|  | Thrombosis |
|  | Dissecting hematoma |
|  | Perforation |
|  | Air embolism |
|  | AV fistula |
