# Supplementary material for: The Effect of Plasma Triglyceride-Lowering Therapy on the Evolution of Organ Function in Early Hypertriglyceridemia-Induced Acute Pancreatitis Patients With Worrisome Features (PERFORM Study): Rationale and Design of a Multicenter, Prospective, Observational, Cohort Study
Source: Front Med (Lausanne). 2021 Dec 13;8:756337. doi: 10.3389/fmed.2021.756337 (PMC8710509; doi:10.3389/fmed.2021.756337)
Supplement: Supplementary file 2 [file Table_2.DOCX]

|  | **EPIDEMIOLOGY** | **OPTIONAL** | **MANDATORY** |  |  |  |  |
| --- | --- | --- | --- | --- | --- | --- | --- |
|  | Age |  | √ |  |  |  |  |
|  | Gender |  | √ |  |  |  |  |
|  |  |  |  |  |  |  |  |
|  | **RISK FACTORS** | **OPTIONAL** | **MANDATORY** |  |  |  |  |
|  | Obesity (body mass index) |  | √ |  |  |  |  |
|  | Hyperlipidemia disease history |  | √ |  |  |  |  |
|  | Hyperlipidemia family history |  | √ |  |  |  |  |
|  | APACHE II score |  | √ |  |  |  |  |
|  |  |  |  |  |  |  |  |
|  | **SYMPTOMS AND PHYSICAL EXAMINATION ON ADMISSION** | **OPTIONAL** | **MANDATORY** |  |  |  |  |
|  | Time of onset of disease |  | √ |  |  |  |  |
|  | Time of enrolment |  | √ |  |  |  |  |
|  | Temperature |  | √ |  |  |  |  |
|  | Mean arterial pressure |  | √ |  |  |  |  |
|  | Heart rate |  | √ |  |  |  |  |
|  | Respiratory rate |  | √ |  |  |  |  |
|  | Mental state |  | √ |  |  |  |  |
|  |  |  |  |  |  |  |  |
|  | **LABORATORY PARAMETERS ON ADMISSION** | **OPTIONAL** | **MANDATORY** |  |  |  |  |
|  | Amylase |  | √ |  |  |  |  |
|  | Lipase |  | √ |  |  |  |  |
|  | Triglyceride (TG) |  | √ |  |  |  |  |
|  | Total cholesterol |  | √ |  |  |  |  |
|  | High density lipoprotein cholesterol (HDL-c) | √ |  |  |  |  |  |
|  | Low density lipoprotein cholesterol (LDL-c) | √ |  |  |  |  |  |
|  | Apolipoprotein A1 (Apo A1) | √ |  |  |  |  |  |
|  | Apolipoprotein B (Apo B) | √ |  |  |  |  |  |
|  | Apolipoprotein E (Apo E) | √ |  |  |  |  |  |
|  | Lipoprotein a [LP(a)] | √ |  |  |  |  |  |
|  | Free fatty acids (FFA) | √ |  |  |  |  |  |
|  | C-reactive protein (CRP) | √ |  |  |  |  |  |
|  | Procalcitonin (PCT) | √ |  |  |  |  |  |
|  | Potassium | √ |  |  |  |  |  |
|  | Sodium | √ |  |  |  |  |  |
|  | Creatinine | √ |  |  |  |  |  |
|  | White blood cell (WBC) | √ |  |  |  |  |  |
|  | Hematocrit | √ |  |  |  |  |  |
|  | Glucose | √ |  |  |  |  |  |
|  | Arterial partial pressure of oxygen | √ |  |  |  |  |  |
|  | Fraction of inspiration O2 | √ |  |  |  |  |  |
|  |  |  |  |  |  |  |  |
|  | **DAILY PHYSICAL EXAMINATION** | **OPTIONAL** | **MANDATORY** |  |  |  |  |
|  | Temperature | √ |  |  |  |  |  |
|  | Mean arterial pressure | √ |  |  |  |  |  |
|  | Heart rate | √ |  |  |  |  |  |
|  | Respiratory rate | √ |  |  |  |  |  |
|  | Mental state |  | √ |  |  |  |  |
|  |  |  |  |  |  |  |  |
|  | **DAILY LABORATORY PARAMETERS** | **OPTIONAL** | **MANDATORY** |  |  |  |  |
|  | Triglyceride (TG) |  | √ |  |  |  |  |
|  | Total cholesterol |  | √ |  |  |  |  |
|  | High density lipoprotein cholesterol (HDL-c) | √ |  |  |  |  |  |
|  | Low density lipoprotein cholesterol (LDL-c) | √ |  |  |  |  |  |
|  | Apolipoprotein A1 (Apo A1) | √ |  |  |  |  |  |
|  | Apolipoprotein B (Apo B) | √ |  |  |  |  |  |
|  | Apolipoprotein E (Apo E) | √ |  |  |  |  |  |
|  | Lipoprotein a [LP(a)] | √ |  |  |  |  |  |
|  | Free fatty acids (FFA) | √ |  |  |  |  |  |
|  | C-reactive protein (CRP) | √ |  |  |  |  |  |
|  | Procalcitonin (PCT) | √ |  |  |  |  |  |
|  | Interleukin 6 | √ |  |  |  |  |  |
|  | Lactate dehydrogenase (LDH) | √ |  |  |  |  |  |
|  | Creatinine |  | √ |  |  |  |  |
|  | Creatine kinase | √ |  |  |  |  |  |
|  | Creatine kinase isoenzyme | √ |  |  |  |  |  |
|  |  |  |  |  |  |  |  |
|  | **DAILY TG-LOWERING THERAPY** | **OPTIONAL** | **MANDATORY** |  |  |  |  |
|  | Insulin dosage |  | √ |  |  |  |  |
|  | Low molecular heparin |  | √ |  |  |  |  |
|  | Plasmapheresis |  | √ |  |  |  |  |
|  |  |  |  |  |  |  |  |
|  | **DAILY CONVENTIONALTHERAPY** | **OPTIONAL** | **MANDATORY** |  |  |  |  |
|  | Enteral nutrition |  | √ |  |  |  |  |
|  | Parenteral nutrition |  | √ |  |  |  |  |
|  | Propofol dosage |  | √ |  |  |  |  |
|  | Mechanical ventilation |  | √ |  |  |  |  |
|  | Renal replacement therapy |  | √ |  |  |  |  |
|  |  |  |  |  |  |  |  |
|  | **ADVERSE EVENTS** | **OPTIONAL** | **MANDATORY** |  |  |  |  |
|  | Hypokalemia | √ |  |  |  |  |  |
|  | Hypocalcemia | √ |  |  |  |  |  |
|  | Immunglobulin depletion | √ |  |  |  |  |  |
|  | Coagulation factor depletion | √ |  |  |  |  |  |
|  | Hypocalcemia | √ |  |  |  |  |  |
|  | Metabolic alkalosis | √ |  |  |  |  |  |
|  | Paresthesia | √ |  |  |  |  |  |
|  | Nausea | √ |  |  |  |  |  |
|  | Vomiting | √ |  |  |  |  |  |
|  | Chest pain | √ |  |  |  |  |  |
|  | Hypotension | √ |  |  |  |  |  |
|  | Tetany | √ |  |  |  |  |  |
|  | Arrhythmias | √ |  |  |  |  |  |
|  | Infection | √ |  |  |  |  |  |
|  | Pain | √ |  |  |  |  |  |
|  | Nerve damage | √ |  |  |  |  |  |
|  | Thrombosis | √ |  |  |  |  |  |
|  | Dissecting hematoma | √ |  |  |  |  |  |
|  | Perforation | √ |  |  |  |  |  |
|  | Air embolism | √ |  |  |  |  |  |
|  | AV fistula | √ |  |  |  |  |  |
|  |  |  |  |  |  |  |  |
|  | **OUTCOMES** | **OPTIONAL** | **MANDATORY** |  |  |  |  |
|  | Daily SOFA score from day1 to day14 |  | √ |  |  |  |  |
|  | Daily SIRS score from day1 to day14 | √ |  |  |  |  |  |
|  | Pancreatic necrosis |  | √ |  |  |  |  |
|  | Septic shock |  | √ |  |  |  |  |
|  | Abdominal hemorrhage |  | √ |  |  |  |  |
|  | Intestinal fistula |  | √ |  |  |  |  |
|  | CT severity index |  | √ |  |  |  |  |
|  | Length of hospitalization |  | √ |  |  |  |  |
|  | Length of ICU stay |  | √ |  |  |  |  |
|  | Severity (mild/moderately severe/severe) |  | √ |  |  |  |  |
|  | Mortality |  | √ |  |  |  |  |
