# Supplementary material for: The Effect of Plasma Triglyceride-Lowering Therapy on the Evolution of Organ Function in Early Hypertriglyceridemia-Induced Acute Pancreatitis Patients With Worrisome Features (PERFORM Study): Rationale and Design of a Multicenter, Prospective, Observational, Cohort Study
Source: Front Med (Lausanne). 2021 Dec 13;8:756337. doi: 10.3389/fmed.2021.756337 (PMC8710509; doi:10.3389/fmed.2021.756337)
Supplement: Supplementary file 3 [file Table_3.DOCX]

List of participating centers

| Number | Hospital |
| --- | --- |
| 01 | Jinjiang Hospital of Traditional Chinese Medicine |
| 02 | The First Affiliated Hospital of Xiamen University |
| 03 | Jinling Hospital, Medical School of Nanjing University |
| 04 | General Hospital of Ningxia Medical University |
| 05 | Fujian Provincial Hospital |
| 06 | Suining Central Hospital |
| 07 | The Second Affiliated Hospital of Chongqing medical university |
| 08 | Qian Xi Nan People’s Hospital |
| 09 | Sir Run Run Shaw Hospital of Zhejiang University School of Medicine |
| 10 | First affiliated hospital of Gannan medical university |
| 11 | The First Hospital of HanDan |
| 12 | Pingxiang People's Hospital |
| 13 | Jining No.1 People’s Hospital |
| 14 | First Affiliated Hospital of Dalian Medical University |
| 15 | The First Affiliated Hospital of Henan University of Science and Technology |
| 16 | The Affiliated Hospital of Zunyi Medical university |
| 17 | Longyan First Affiliated Hospital of Fujian Medical University |
| 18 | First Hospital of Jilin University |
| 19 | The Second Affiliated Hospital, Anhui Medical University |
| 20 | Emergency Department of Shangqiu First People's Hospital |
| 21 | Zhengzhou Central Hospital affiliated to Zhengzhou University |
| 22 | The Second Xiangya Hospital of Central South University |
| 23 | Affiliated Hospital of Southwest Medical University |
| 24 | West China Hospital, Sichuan University |
| 25 | Shangrao people’s Hospital |
| 26 | Affiliated Hospital of Integrated Traditional Chinese and Western Medicine, Nanjing University of Chinese Medicine |
| 27 | The NO. 909 Hospital of Chinese PLA joint Logistics support Force |
| 28 | Dazhou Central Hospital |
